# *AtCTF7* is required for establishment of sister chromatid cohesion and association of cohesin with chromatin during meiosis in Arabidopsis

**DOI:** 10.1186/1471-2229-13-117

**Published:** 2013-08-14

**Authors:** Dipesh K Singh, Sebastien Andreuzza, Aneesh P Panoli, Imran Siddiqi

**Affiliations:** 1 Centre for Cellular & Molecular Biology (CSIR), Uppal Road, Hyderabad 500007, India

**Keywords:** Plant meiosis, Chromosome organization, Synapsis, Conditional RNAi, Gametogenesis, DNA repair

## Abstract

**Background:**

The establishment of sister chromatid cohesion followed by its controlled release at the metaphase to anaphase transition is necessary for faithful segregation of chromosomes in mitosis and meiosis. Cohesion is established by the action of Ctf7/Eco1 on the cohesin complex during DNA replication following loading of cohesin onto chromatin by the Scc2-Scc4 complex. Ctf7 is also required for sister chromatid cohesion during repair of DNA double strand breaks. Ctf7 contains an acetyltransferase domain and a zinc finger motif and acetylates conserved lysine residues in the Smc3 subunit of cohesin. In Arabidopsis CTF7 is encoded by a single gene and mutations in *AtCTF7* cause embryo lethality indicating that the gene is essential.

**Results:**

To study the function of Ctf7 in plants and to determine its role in sister chromatid cohesion, we constructed a conditional allele of *AtCTF7* in Arabidopsis using an inducible RNA interference (RNAi) strategy, so as to avoid the embryo lethality caused by mutations in *AtCTF7*. We found that induction of RNAi against *AtCTF7* caused severe inhibition and defects in growth during vegetative and reproductive stages as well as sterility. *AtCTF7*-RNAi plants displayed chromosome fragmentation and loss of sister chromatid cohesion during meiosis. Immunostaining for the cohesion subunit AtSCC3 showed a marked reduction in association of cohesin with chromatin during meiosis in *AtCTF7*-RNAi plants.

**Conclusions:**

We find that *AtCTF7* is essential for sister chromatid cohesion during meiosis in Arabidopsis and is required for association of cohesin with chromatin in prophase of meiosis.

## Background

Proper chromosome segregation during cell division requires that sister chromatids produced by DNA replication are held together until their controlled separation at anaphase. This function is accomplished by the cohesin complex, whose conserved core subunits consist of the Structural Maintenance of Chromosome (SMC) proteins Smc1 and Smc3, the Sister Chromatid Cohesion (SCC) protein Scc3, and the α-kleisin protein Scc1 [1]. According to the ring model of cohesin action, Smc1 and Smc3 interact to form a V shaped heterodimer, closed by Scc1 with the help of Scc3, to form a ring that is considered to entrap sister chromatids and hold them physically together [2,3]. Cohesion is released at anaphase by the cleavage of Scc1 by separase, a protease that is activated by the anaphase promoting complex/cyclosome (APC/C) [3].

In *Saccharomyces cerevisiae*, cohesion is established by Ctf7/Eco1, after cohesin has been loaded on chromatin by the Scc2-Scc4 complex [4-6]. Ctf7 establishes cohesion during S phase, and interacts with components of the DNA replication machinery, including PCNA and RFC [5-7]. These results led to a model in which sister chromatid cohesion is established concomitantly with DNA replication [8]. Ctf7 encodes a zinc finger protein with an active acetyltransferase domain, and it was found that Ctf7 acetylation of Smc3 on conserved lysines, was critical for establishment of cohesion by counteracting the Wpl1-Pds5 complex in preventing establishment of cohesion [9-14]. Establishment of cohesion has been suggested to occur in concert with lagging strand synthesis [15], and Smc3 acetylation leading to establishment of functional cohesion occurs only in association with replication [16]. Recycling of the Smc3 subunit is aided by deacetylation by Hos1 following cleavage of Scc1 by separase to release cohesion at the metaphase to anaphase transition, and is important for establishment of cohesion [17-19]. The *eso1-H17* mutant in *Schizosaccharomyces pombe* exhibits delayed mitosis as a result of activation of the spindle checkpoint, and defective segregation of chromosomes in mitosis [20]. In *Drosophila*, mutations in Deco result in altered distribution of cohesin at metaphase, and premature entry into anaphase [21]. In humans, mutations in *ESCO2* cause Roberts syndrome which results from a deficiency of cohesion around the centromeres, and encompasses a number of developmental abnormalities as well as mental retardation and renal and cardiac dysfunction [22].

The machinery for establishment of cohesion is conserved in Arabidopsis, and homologues of Scc2 and Ctf7 have been identified and functionally characterized. Mutations in *AtSCC2* and *AtCTF7* result in embryo lethality, however *AtCTF7* is dispensable for endosperm growth [23,24]. Interestingly, *AtCTF7* was found to possess acetyltransferase activity *in vitro*, and could complement the yeast *ctf7-203* mutant, suggesting conserved biochemical function with its yeast counterpart [23]. By using a conditional RNA interference (RNAi) approach, it has been demonstrated that *AtSCC2* is required during meiosis for sister chromatid cohesion, chromosomal axis formation and synapsis between homologues [24]. The function of *AtCTF7* in establishment of sister chromatid cohesion *in planta* remains to be shown.

Here, we used a conditional RNAi approach to examine the role of *AtCTF7* in sister chromatid cohesion, and to analyze the effects of the loss of *AtCTF7* during vegetative and reproductive development. We found that downregulation of *AtCTF7* severely inhibited growth during vegetative and reproductive stages, and resulted in both male and female sterility. During meiosis, *AtCTF7*-RNAi lines displayed typical loss of cohesion phenotypes, including abnormal chromosome organization, impaired chromosome synapsis and DNA fragmentation. Consistent with an expected involvement of *AtCTF7* in cohesion, we found that sister chromatid cohesion was lost at both chromosome arms and centromeres in *AtCTF7*-RNAi plants. Finally, we found that AtSCC3 localization on chromatin was compromised during meiosis in *AtCTF7*-RNAi plants, indicating that *AtCTF7* is required for association of cohesin with chromosomes in Arabidopsis, a feature that appears to be similar to Drosophila, where Deco is required for Scc1 association with chromosomes during M phase [21]. Overall, our results establish an essential role for *AtCTF7* in vegetative development and in sister chromatid cohesion during meiosis.

## Results

### AtCTF7 is required for growth during vegetative and reproductive stages

**Figure 1:**
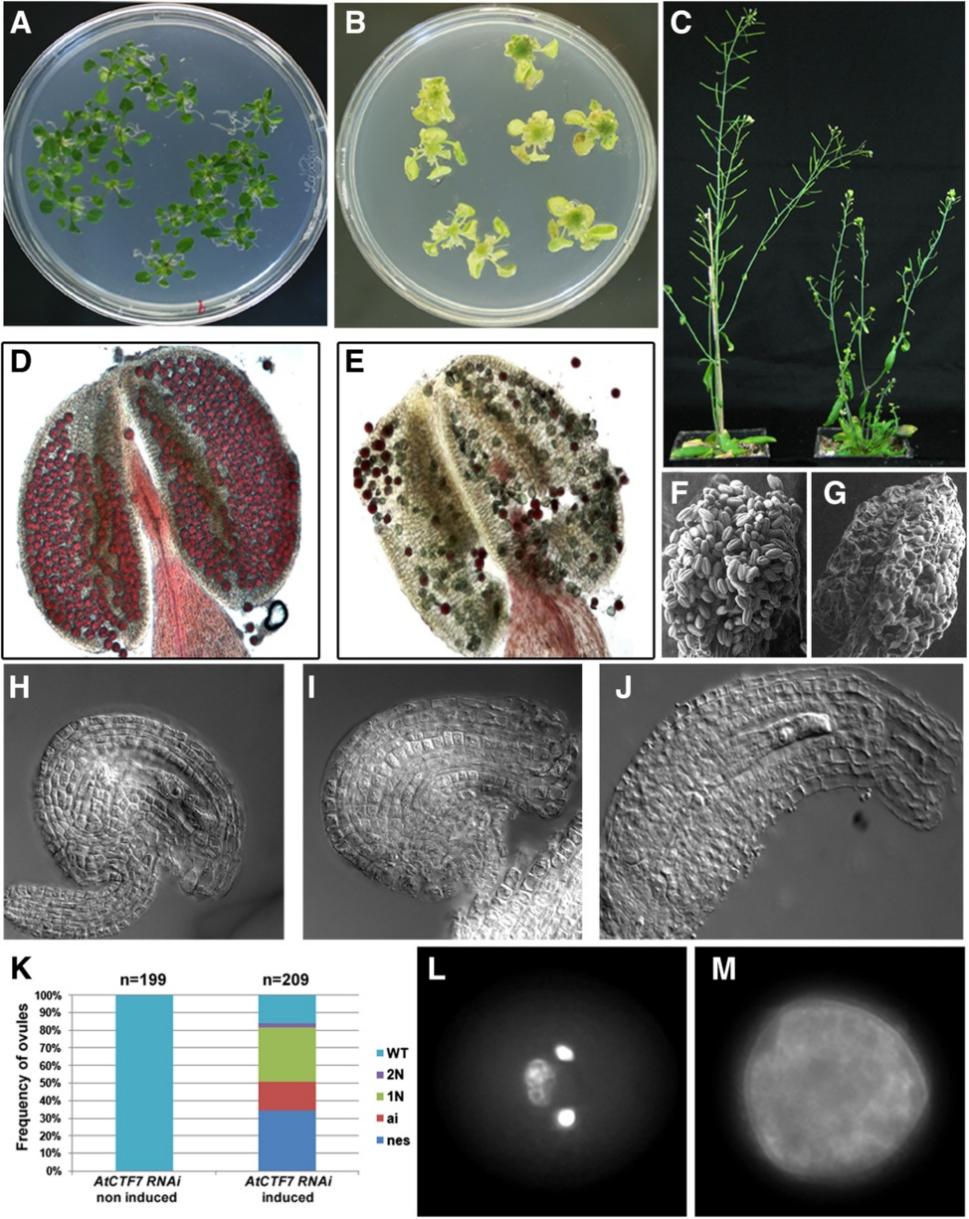
**Knockdown of AtCTF7results in growth defects and sterility.** The RNAi mediated down-regulation of AtCTF7 results in growth inhibition, female and male gametophyte defects, and sterility. **(A,B)** Loss of coloration and growth inhibition in treated **(B)** compared to untreated **(A)***AtCTF7-*RNAi seedlings. **(C)** Impaired growth and sterile siliques in treated (right) compared to untreated (left) *AtCTF7-*RNAi adult plants. **(D,E)** Alexander staining of mature anthers shows loss of pollen viability (green grains instead of purple) in treated **(E)** compared to untreated **(D)***AtCTF7-*RNAi mature anthers. **(F,G)** Scanning electron micrograph of anther from untreated **(F)** and treated **(G)***AtCTF7-*RNAi plant showing reduction of pollen grains following treatment. **(H-J)** Impaired female gametophyte and ovule development in treated **(I,J)** compared to untreated **(H)***AtCTF7-*RNAi plants. **(I)** Ovule lacking a female gametophyte. **(J)** Ovule with abnormal integument development. **(K)** Quantification of female gametophytic and ovule defects shown in **(I)** and **(J)** for ovule stages 3–4 to 3–6 [26]. Gametophytes were classified as uninucleate (1N), binucleate (2N), missing (nes), or wild type (WT) if they contained greater than 2 nuclei. ai: ovules showing abnormal integument development. **(L,M)** DAPI staining of mature pollen grains reveals loss of chromatin in the male gametophyte of treated **(M)** compared to untreated **(L)***AtCTF7-*RNAi plants, which contains two sperm cells and one vegetative cell.

**Figure 2:**
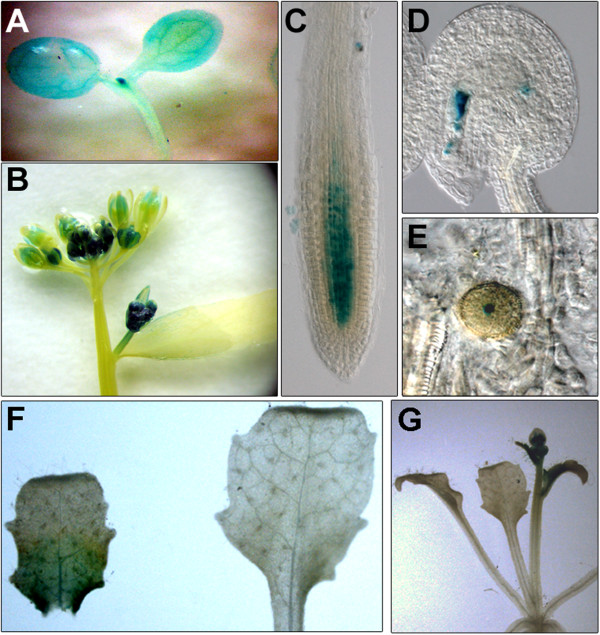
**AtCTF7 is expressed in actively dividing tissues.** Examination of a *P*_*AtCTF7*_*nlsGUS* reporter shows expression in dividing tissues. **(A)** Shoot meristematic region and cotyledonary leaves. **(B)** Inflorescence and young buds. **(C)** Root meristem. **(D)** Female gametophyte. **(E)** Pollen. **(F)** Young cauline leaf (left) and expanded rosette leaf (right). **(G)** Portion of whole plant showing GUS expression in inflorescence but not in rosette leaves.

To examine the requirement for AtCTF7 during different stages of development we constructed a conditional allele using an inducible RNAi approach to deplete AtCTF7 mRNA. A hairpin RNAi construct for *AtCTF7* was made and placed under control of the dexamethasone-inducible transactivator LhGR [25]. T1 transformants were grown and seeds collected. T2 plants were then grown and treated with dexamethasone either at the vegetative stage or after bolting. Treatment during the vegetative stage resulted in defective growth in both aerial and root tissues, accompanied by loss of greening (Figure 1A and B, Additional file 1: Figure S1), indicating that AtCTF7 is required for plant growth during vegetative stages. Treatment with dexamethasone following bolting resulted in strong inhibition of growth of the inflorescence as well as sterility (Figure 1C). Pollen was largely sterile and inviable following dexamethasone treatment (Figure 1D-G), and lacked clearly defined sperm and vegetative nuclei (Figure 1L and M, Additional file 1: Figure S2). Ovules in treated plants showed arrest in female gametogenesis starting at an early stage (1n) as well as missing embryo sacs (Figure 1H-K). A proportion of ovules also showed defects in integument development (Figure 1J). A total of 29 independent transformants were examined, out of which 5 lines showed strong growth defects and sterility following treatment with dexamethasone. Quantitative comparison of AtCTF7 expression indicated a reduction in treated compared to untreated control plants (Additional file 1: Figure S3), consistent with the growth defects being due to depletion of *AtCTF7*. Examination of *AtCTF7* promoter activity using a *P*_*AtCTF7*_*nlsGUS* reporter indicated that the *AtCTF7* promoter is active in both the shoot and root meristematic regions (Figure 2A and 2C), as well as in young buds and leaves (Figure 2A and 2B). Expression is reduced in older leaves and flowers (Figure 2B). Within reproductive cells, expression was observed in pollen and in the female gametophyte (Figure 2D and 2E), and is consistent with the defects in gametogenesis described above for *AtCTF7*-RNAi plants.

These results indicate that AtCTF7 is expressed in dividing cells and is required for normal development and growth during both vegetative and reproductive stages.

### Knockdown of AtCTF7 results in defects in sister chromatid cohesion and chromosome organization during meiosis

**Figure 3:**
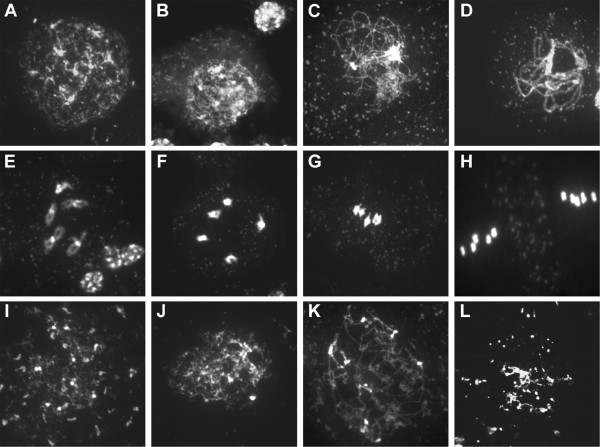
**Defective meiotic chromosome organization in *AtCTF7-*RNAi.** Acid spreads of male meiotic chromosomes stained with DAPI. **(A-H)** Untreated *AtCTF7-*RNAi line, **(I-L)***AtCTF7-*RNAi line treated with dexamethasone. **(A)** Meiotic interphase. **(B)** Leptotene. **(C)** Zygotene. **(D)** Pachytene showing complete chromosome synapsis. **(E)** Late diplotene showing 5 partially condensed bivalents. **(F)** Diakinesis showing 5 condensed bivalents. **(G)** Metaphase I showing 5 aligned bivalents. **(H)** Metaphase II showing two sets of 5 aligned chromosomes. **(I)** Early prophase stage showing few thread-like segments and many discontinuities. **(J)** Early prophase stage with discontinuous and fragmented chromatin. **(K)** Mid-prophase showing unpaired, discontinuous chromatin and absence of synizetic knot. **(L)** Late prophase stage, showing condensed and fragmented chromatin.

To examine whether AtCTF7 is required in meiosis, chromosome spreads were carried out on male meiocytes from *AtCTF7*-RNAi plants (Figure 3). Plants that had not been treated with dexamethasone showed normal pairing and synapsis, and normal organization of meiotic chromosomes (Figure 3A-H). Early prophase stages were marked by the appearance of thin chromosome threads at leptotene, followed by zygotene and pachytene stages showing normal pairing and synapsis of chromosomes (Figure 3A-D). Subsequent late prophase stages and meiosis I and II stages, were also seen to occur normally in untreated plants (Figure 3E-H). In contrast, plants that were treated with dexamethasone showed severe defects in meiotic chromosome organization (Figure 3I-L). The chromosomal defects were apparent early in prophase I for which the characteristic stages could not be clearly distinguished. Chromosomes appeared disorganized and had a patchy appearance with discontinuities. The synizetic knot did not form and mid to late prophase stages were marked by chromosomes appearing clumped and highly fragmented (Figure 3I-K). Late prophase stages were characterized by the presence of condensed fragments of chromosomes which could exceed 20 in number indicating that fragmentation had taken place (Figure 3L).

**Figure 4:**
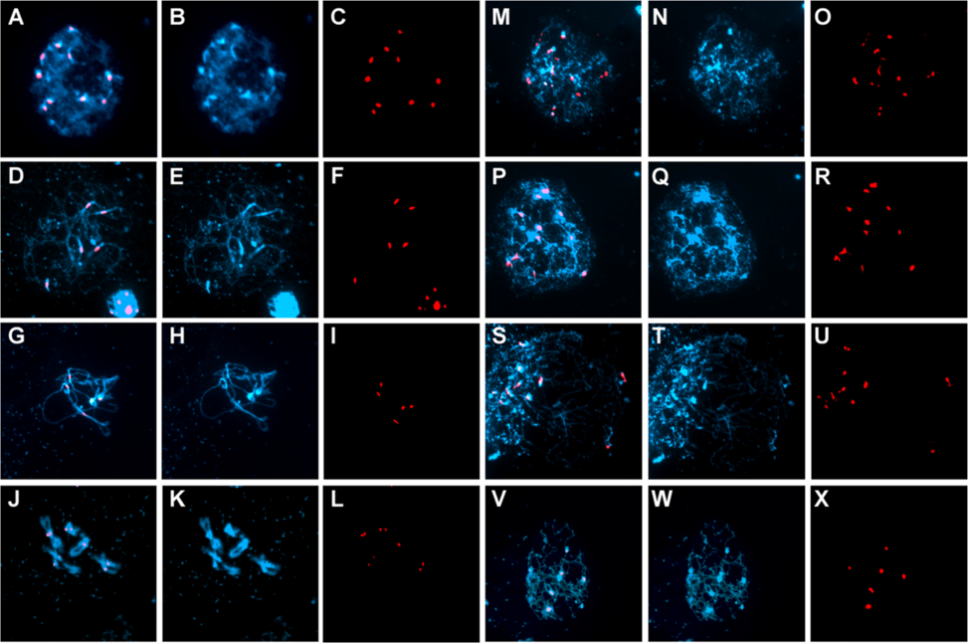
**Loss of centromeric pairing and cohesion in *AtCTF7*-RNAi male meiocytes.** FISH of male meiotic chromosome spreads hybridized with a centromeric probe showing DAPI (blue) and probe (red). **(A-L)** Untreated transgenic *AtCTF7-*RNAi line, **(M-X)** transgenic *AtCTF7-*RNAi line treated with dexamethasone. For each panel, left column shows merged images of DAPI and the probe, the middle column shows DAPI, and the right column shows the probe alone. **(A-C)** Early leptotene cell showing 10 centromeres. **(D-F)** Late zygotene showing 5 centromeric signals as a result of synapsis of centromeres. **(G-I)** Pachytene showing 5 centromeric signals as synapsis of chromosomes is complete. **(J-L)** Diplotene. **(M-O, P-R)** Early meiotic prophase, showing more than 10 centromeric signals. **(S-U, V-X)** Mid meiotic prophase stages, showing more than 10 centromeric signals **(S-U)** and 6 centromeric signals **(V-X)**.

**Figure 5:**
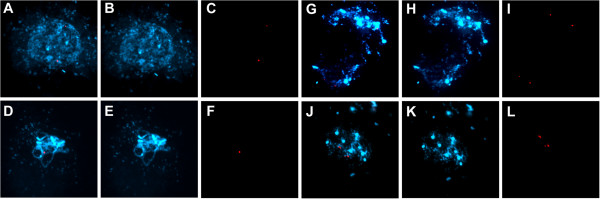
**Loss of chromosome arm cohesion and impaired synapsis during male meiosis in *AtCTF7-*RNAi.** FISH of male meiotic chromosome spreads hybridized with a chromosome 4 arm probe showing chromosomes stained with DAPI (blue) and probe (red). **(A-F)** Untreated transgenic *AtCTF7-*RNAi line; **(G-L)** transgenic *AtCTF7-*RNAi line treated with dexamethasone. For each set, the left image shows DAPI and the probe merged images, the middle image shows DAPI, and the right image shows the probe alone. **(A-C)** Early leptotene showing 2 signals. **(D-F)** Pachytene showing a single probe signal due to complete synapsis of chromosomes. **(G-I)** Early prophase showing 4 signals, indicating loss of chromatid cohesion. **(J-L)** Mid-prophase stage showing 3 signals.

To examine the role of AtCTF7 in sister chromatid cohesion and pairing at the centromeric region, we carried out fluorescence in situ hybridization (FISH) using a centromere repeat probe that hybridizes to all the centromeres [24]. In untreated control plants, 8–10 centromere signals (mean = 9.5; n = 33) could be clearly detected at early prophase stages (leptotene) in meiocytes, whereas at zygotene and pachytene stages, 3–5 signals (mean = 3.1; n = 72) were observed (Figure 4A-L). For dexamethasone treated plants, the number of signals at early prophase ranged between 9 and 20 (mean = 12.0; n = 64). The presence of greater than 10 signals at prophase I indicated loss of centromere cohesion in *AtCTF7*-RNAi plants (Figure 4M-X). In addition there was variability in shape of the signal in *AtCTF7*-RNAi plants compared to untreated control plants which suggested defects in centromere organization. These results indicate that AtCTF7 is required for pairing, cohesion, and proper organization of centromeric regions. To examine arm cohesion we used a BAC probe specific for chromosome 4 (Figure 5). In untreated plants, we observed two signals in early prophase, and a single signal in meiocytes at mid-prophase stages when chromosomes had fully synapsed (Figure 5A-F), whereas in plants that were treated with dexamethasone, we observed more than two signals (Figure 5G-L). Overall the results indicate that AtCTF7 is required for both centromere and arm cohesion.

### Reduced association of cohesin with chromatin in *AtCTF7*-RNAi plants

**Figure 6:**
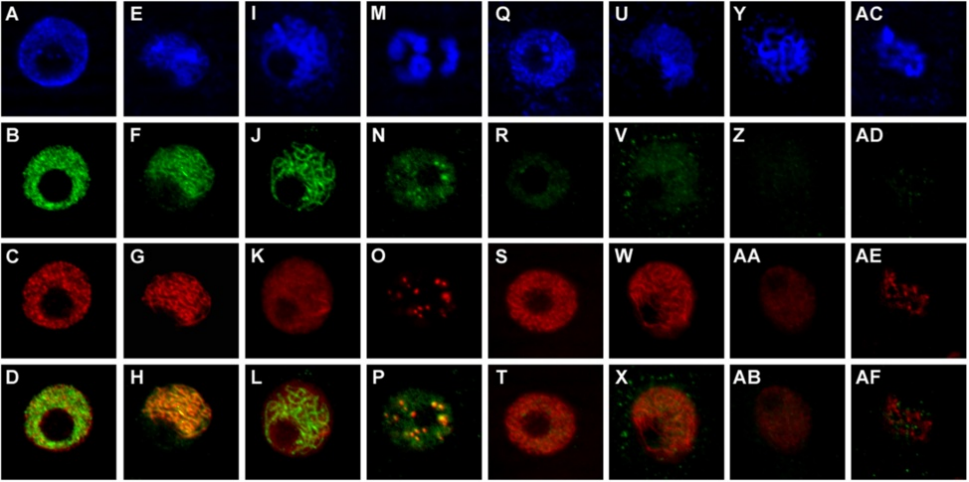
**Impaired loading of AtSCC3 cohesin on meiotic chromosomes in *AtCTF7-*RNAi.** Immunostaining of anther squashes showing chromosomes stained with DAPI (blue), AtSCC3 (green), ASY1 (red) and merged images for the green and red channels (bottom row). **(A-P)**, untreated transgenic *AtCTF7-*RNAi line, **(Q-AF)** transgenic *AtCTF7-*RNAi line treated with dexamethasone. **(A-D, Q-T)** Early leptotene. **(E-H, U-X)** Late leptotene. **(I-L, Y-AB)** Pachytene. **(M-P, AC-AF)** Diplotene.

In order to examine the role of AtCTF7 in sister chromatid cohesion and chromosome organization,we examined the localization of the cohesin subunit AtSCC3 [27], and the axial element protein ASY1 [28] during male meiosis (Figure 6, Additional file 1: Figure S4). In untreated control plants, AtSSC3 immunostaining extended throughout the chromatin at early prophase stages, and marked chromosomal axes during zygotene and pachytene stages (Figure 6A-P). In contrast, the level of AtSCC3 immunostaining, was greatly reduced in male meiocytes from *AtCTF7*-RNAi plants from early prophase onwards (Figure 6Q-AF). ASY1 immunostaining in control plants is seen at early prophase and marks the chromosome axes at leptotene (Figure 6C, G, K, and O). The staining pattern overlaps with that of AtSCC3 (Figure 6D, H, L, P). In the case of *AtCTF7*-RNAi plants, association of ASY1 with chromatin was comparable to that of control plants (Figure 6S, W, AA, AE). Thus, although ASY1 staining of chromatin shows significant overlap with that of AtSCC3, its association with chromatin appears to be independent of AtSCC3 which is known to precede ASY1, and is also consistent with analysis of the *Atscc3-1* mutant [27].

## Discussion

Ctf7/Eco1 proteins have been shown to control establishment of cohesion in yeast, *Drosophila*, and mammals [5,6,20,21,29]. In the case of plants, the Arabidopsis homolog of Ctf7 (AtCTF7) has been shown to be required for embryo development but not required for development of the endosperm [23], leaving open the possibility of a Ctf7-independent mechanism for sister chromatid segregation operating in meiosis. Evidence for a Ctf7-independent mechanism for sister chromatid segregation in yeast is based on the viability of an *eco1∆ wpl1∆*strain [30]. A dosage dependent role for Ctf7 in meiosis in yeast has been suggested based on haplo-insufficiency during sporulation [15]. Establishment of sister chromatid cohesion in meiosis in yeast may therefore be more sensitive to reduced dosage of Ctf7 than in mitosis. Alternatively, acetylation of other proteins by Ctf7 during meiosis may also be involved [31]. A role for Ctf7 in meiosis is also suggested from an examination of the localization and regulation of murine ESCO2 [32], however a requirement for Ctf7 in sister chromatid cohesion specifically during meiosis remains to be established. In this study we have shown using Arabidopsis as a model, that AtCTF7 is also required for sister chromatid cohesion in meiosis.

The conditional RNAi approach to examine the function of AtCTF7 in plants revealed defects in sister chromatid cohesion in meiosis. The establishment of sister chromatid cohesion in both arm and centromeric regions during meiosis was dependent upon AtCTF7. The meiotic phenotypes comprised defects early in prophase which presented as discontinuities in the thread-like appearance characteristic of leptotene and zygotene stages. In the most severe cases, the thread-like structure was largely absent and the chromatin appeared highly fragmented. At later prophase stages, the fragmented phenotype was further apparent by the presence of a large number of separated and condensed chromatin fragments. The results are consistent with a failure (in meiosis) to repair double strand breaks for which Ctf7 is known to be required [33]. The fragmentation phenotype is similar to that observed for *Atrec8* and *Atmnd1* mutants, which are defective in repair of meiotic double strand breaks [34,35]. However, since the RNAi strategy employed is not specific to meiosis, the possibility that the fragmentation phenotype may also be influenced by depletion of AtCTF7 earlier during mitosis in the progenitor cells of the meiocytes is not ruled out. Arabidopsis mutants defective in both cohesion and formation of meiotic double strand breaks do not display such fragmentation phenotypes [27,36].

We observed severe defects in vegetative as well as reproductive growth and development, pointing to a role for *AtCTF7* throughout the plant life cycle, and extending previous work showing an essential requirement for *AtCTF7* in embryo development [23]. A *P*_*AtCTF7*_*nlsGUS* reporter was strongly expressed in root and shoot meristems, and in young buds and leaves. In young developing leaves, a polarity in expression was observed with GUS staining confined to the basal part of the leaf and absent towards the distal portion. The gradient of expression is similar to that for the cell division marker CycB1;1::GUS [37]. Expression declined in older buds and was not observed in expanded rosette leaves. The expression of AtCTF7 is thus seen to occur in tissues that are undergoing active cell division, consistent with the known involvement of Ctf7 in promoting establishment of cohesion in conjunction with DNA replication [8]. Within reproductive cells, expression was observed in pollen and in the female gametophyte. The sterile phenotype we observed is likely to be accounted for mainly by the defects in meiosis as well as a possible contribution from a gametophytic component.

Establishment of cohesion by Ctf7 involves acetylation of conserved lysine residues in the Smc3 subunit of cohesion which inhibits the action of the Wpl1-Pds5 complex in preventing establishment of cohesion [9,10,12-14]. In yeast, *Drosophila*, and human cells, Ctf7/Eco1 is required for the establishment of cohesion but not for association of cohesin with chromatin in interphase [5,21,29]. In *Drosophila*, a *deco* mutant shows reduced staining for the cohesin subunit Scc1 at prometaphase of mitosis [21]. The strong reduction in association of AtSCC3 with chromatin in early meiotic prophase as revealed by immunostaining of meiocytes in *AtCTF7*-RNAi plants is similar to what has been observed for the *deco* mutant in prometaphase of mitosis, and suggests conservation of Ctf7/Eco1 function in plants.

## Conclusions

In conclusion, our findings show that AtCTF7 is required for establishment of sister chromatid cohesion during meiosis in Arabidopsis, and that continued association of cohesin with chromatin in meiosis depends on AtCTF7.

## Methods

### Plant materials and growth conditions

The *Arabidopsis thaliana* strains used were of the Columbia ecotype (Col-0). Plants were grown as described in [38]. To generate transgenic Arabidopsis, constructs were mobilized into *Agrobacterium tumefaciens* strain AGL-1 using triparental mating, and transformed into *Arabidopsis* by vacuum infiltration as described in [39]. *P*_*AtCTF7*_*nlsGUS* was transformed into wild-type Col, and *AtCTF7*-RNAi was transformed into a line carrying a *P*_*CaMV35S*_*LhGR-N* transgene [25]. Transgenic plants were selected on MS media, containing 120 μg/ml gentamycin (Sigma-Aldrich) for *AtCTF7*-RNAi transformants, and 50 μg/ml kanamycin for *P*_*AtCTF7*_*nlsGUS* transformants, and were further confirmed by PCR.

### Cloning procedures

The *AtCTF7* promoter, comprising 646 bp upstream of the ATG and 45 bp from *AtCTF7* coding sequence, was amplified by PCR using primers Fctf7gusHindIII and Rctf7gusnlsBamH1, and cloned as a BamH1-HindIII fragment in frame with a *nlsGUS* tag in the pBI101.2 binary vector. For the RNAi construct, 658 bp fragments were amplified by PCR using primer pairs F1rnaiXba1 and R1rnaiBamH1, and F1rnaiXho1 and R1rnaiEcoR1, and cloned as Xba1-BamH1 and Xho1-EcoR1 fragments in opposite directions in pKANNiBAL [40]. The RNAi cassette was excised as a XhoI-BamH1 fragment and cloned into the binary vector pZP222-6xPOP described in [25].

### Dexamethasone treatment

Transgenic seeds were germinated on MS plates, and grown for 7 days after which they were transferred on MS plate containing 20 μM dexamethasone (Sigma). Seedlings were analyzed for phenotypes 7 days after transfer on dexamethasone plates. Treatment of adult plants after bolting was carried out by inclusion of 20 μM dexamethasone in the watering solution which was delivered by subirrigation. Samples for meiotic analysis were collected for analysis 5 days after the start of dexamethasone treatment.

### RNA isolation and quantitative RT-PCR

Total RNA was isolated using Trizol (Invitrogen) following the manufacturer’s protocol. cDNA synthesis was performed using Reverse Transcription System (Invitrogen SuperScript II) and oligo(dT) primers. Real Time PCR reactions were performed using SYBR Green PCR master mix (Applied Biosystems). GAPC was used as the internal normalization control. PCR was performed on the ABI Prism 7900 HT Sequence Detection System (Applied Biosystems) in a 384 well reaction plate according to the manufacturer’s recommendations. Primers used were Ctf7qRTF and Ctf7qRTR for *AtCTF7*, and GAPRTF and GAPRTR for GAPC (Additional file 1: Table S1). Cycling parameters consisted of 2 minutes incubation at 50°C, 10 minutes at 95°C, and 40 cycles of 95°C for 15 seconds, 57°C for 30 seconds and 67°C for 30 seconds. The PCR reaction was performed in triplicate for each RNA sample, and the experiment was carried out on two different biological samples representing the same RNAi line. Specificity of the amplifications was verified at the end of each PCR run using ABI prism dissociation curve analysis software. Results from the ABI Prism 7900 HT Sequence Detection System were analyzed further using Microsoft Excel. Relative amounts of mRNA were calculated from threshold points (Ct values) located in the log-linear range of real time PCR amplification plots using the 2-ΔCt method. Standard deviations in Additional file 1: Figure S1 are for variation across biological samples.

### Cytological procedures

Whole mount analysis of ovules was done after fixing and clearing the inflorescence in methyl benzoate as described previously [38]. Scanning electron microscopic (SEM) analysis of pollen was carried out using a Hitachi scanning electron microscope (model 3400 N, http://www.hitachi-hitec.com). Pollen viability was examined using Alexander staining [41]. For DAPI analysis of pollen, anthers were squashed and stained with DAPI (1 μg/ml). Meiotic chromosome spreads were carried out as described in [42], with minor modifications [43]. Observations were made on a Zeiss Axioplan 2 imaging microscope, using a Plan Apochromat 63 × oil immersion objective. Tissue from *P*_*AtCTF7*_*nlsGUS* transgenic plants was stained for GUS activity as described in [38].

For FISH, chromosome spreads were carried out as described above, and FISH analysis was carried out according to the method described in [44], with minor modifications [35]. The 180-bp centromeric pAL1 repeat was used to detect centromere sequences [45]. A plasmid harboring two copies of the pAL1 repeat was subjected to PCR in the presence of Cy3-dATP (GE Healthcare), using PAL forward and reverse primers (Additional file 1: Table S1). BAC clones T19F6 and T22A6 from chromosome 4 were used as probes to monitor arm cohesion after being subjected to nick translation and labeling by Cy3-dATP (Roche). Slides were observed under a Zeiss Axioplan 2 imaging microscope equipped with a Plan Apochromat 63× oil immersion objective, using an excitation (Cy3, 550 nm) and long-pass emission (Cy3, 570 nm) filter.

For immunostaining, inflorescences were fixed as described in [46]. Young buds were dissected out and washed with 10 mM Citrate Buffer pH4.5 (1× CB), followed by digestion with a cell wall digesting enzyme mix containing 0.3% cellulase, 0.3% pectolyase, 0.4% cytohelicase (all Sigma) in 1× CB, and incubated for 30 min at 37°C. The enzyme mix was replaced with 1× PBS, and anthers were dissected out from buds on a slide and squashed using a 22×22 mm coverslip. The slide was snap-frozen by dipping in liquid nitrogen and the coverslip was immediately removed. Slides were then dried and dipped briefly in molten 1% gelatin, 1% agarose solution to cover the cells with a thin layer of gelatin-agarose and dried. Slides were rehydrated in 1× PBS, and digested with the enzyme mix described above for 30 min at 37°C. This was followed by permeabilization of the cells in 1× PBS, 1% Triton-X100, for 30 min and washing of the slides 2–3 times in 1× PBS containing 0.1% Triton-X100. Immunostaining was performed as described in [24], using ASY1 antibody at a 1:1000 dilution, and AtSCC3 antibody at 1:200 dilution. All secondary antibodies were used at a dilution of 1:100. Slides were mounted in 1ug/ml DAPI in Vectashield (VectorLabs). Cells were imaged using a Zeiss Axio Imager.Z1 microscope equipped with an apotome module, using a Plan-Apochromat 63× oil-immersion objective.

While this manuscript was under review, a related study appeared online by Bolanos-Villegas et al., on the role of AtCTF7 in DNA repair, mitosis, and meiosis [47].

## Competing interests

The authors declare that they have no competing interests in the work presented in this study.

## Authors’ contributions

IS, DS, and AP designed the experiments. DS performed the experiments with contributions from SA. DS, IS, and SA prepared the manuscript. All authors read and approved the final manuscript.

## Supplementary Material

Additional file 1: Figure S1 Effect of dexamethasone treatment on wild type. **Figure S2.** Microscopic analysis of pollen in *AtCTF7-*RNAi. **Figure S3.** Quantitative reverse transcription PCR (q-RT-PCR) of AtCTF7 in *AtCTF7*-RNAi. **Figure S4.** Raw pictures of AtSCC3 and ASY1 immunostaining on untreated and treated *AtCTF7*-RNAi line. **Table S1.** List of primers used. Click here for file
