# Supplementary material for: AtCTF7 is required for establishment of sister chromatid cohesion and association of cohesin with chromatin during meiosis in Arabidopsis
Source: BMC Plant Biol. 2013 Aug 14;13:117. doi: 10.1186/1471-2229-13-117 (PMC3751900; doi:10.1186/1471-2229-13-117)
Supplement: Additional file 1: Figure S1 — Effect of dexamethasone treatment on wild type. Figure S2. Microscopic analysis of pollen in AtCTF7-RNAi. Figure S3. Quantitative reverse transcription PCR (q-RT-PCR) of AtCTF7 in AtCTF7-RNAi. Figure S4. Raw pictures of AtSCC3 and ASY1 immunostaining on untreated and treated AtCTF7-RNAi line. Table S1. List of primers used. [file 1471-2229-13-117-S1.docx]

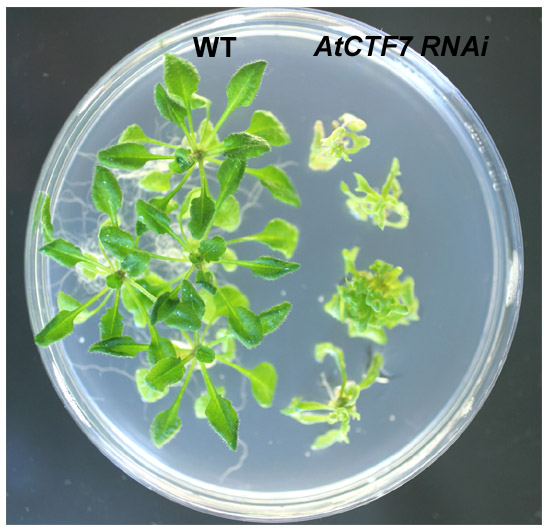


**Supplementary Figure 1: Effect of dexamethasone treatment on wild type.** Wild type and *AtCTF7*-RNAi seedlings grown side by side as indicated on 20 μM dexamethasone plate. Severe growth defects are seen in the RNAi line whereas wild type grows normally.


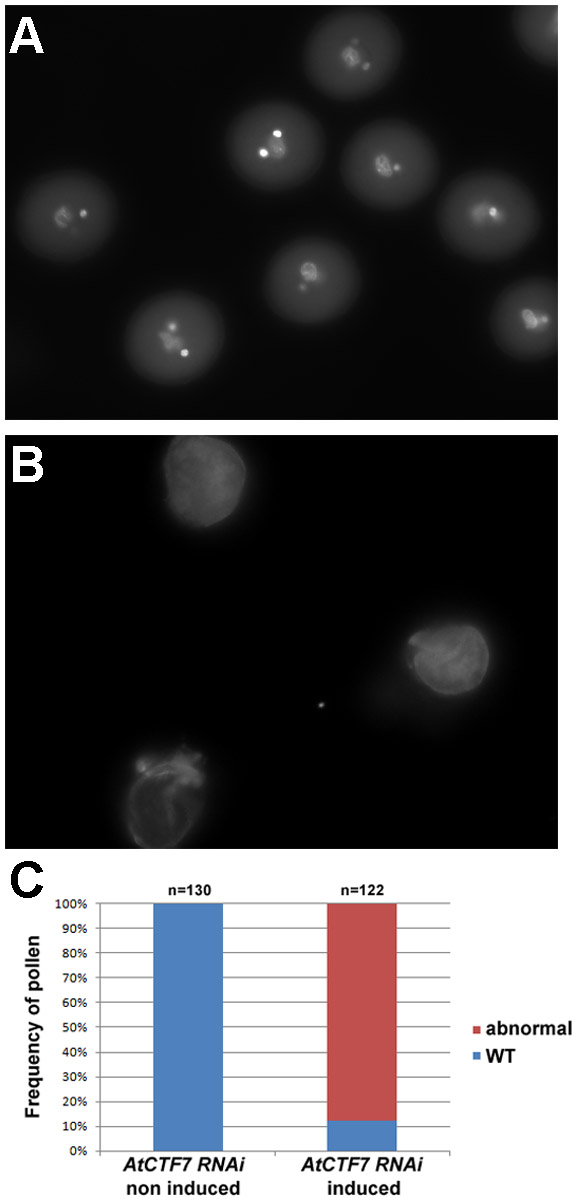


**Supplementary Figure 2: Microscopic analysis of pollen in *AtCTF7-*RNAi.** DAPI staining of mature pollen from uninduced (**A**) and induced (**B**) *AtCTF7*-RNAi. (**C**) Quantification of the observed pollen phenotypes in *AtCTF7*-RNAi.

**
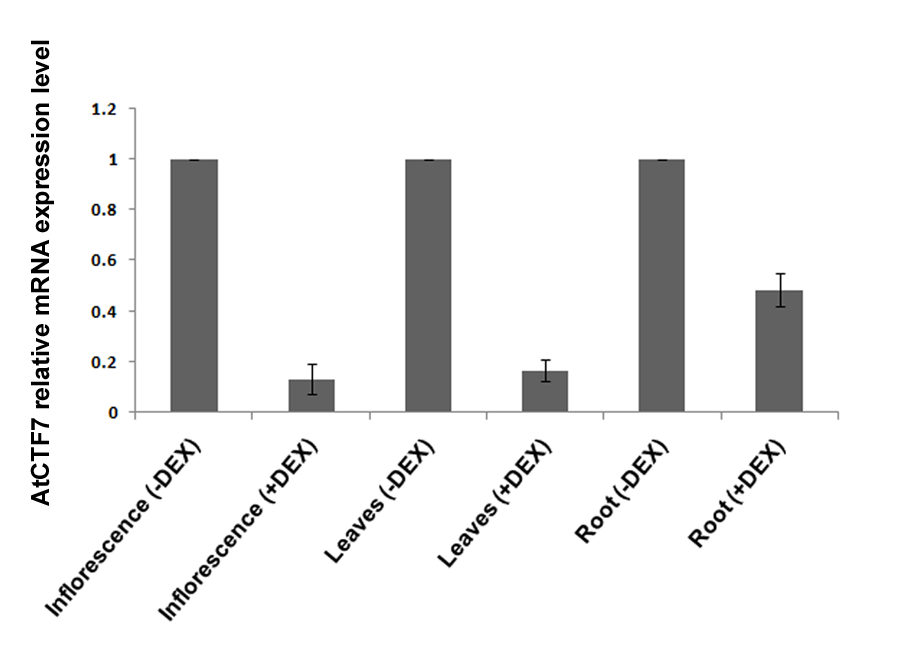
**

**Supplementary Figure 3: Quantitative reverse transcription PCR (q-RT-PCR) of AtCTF7 in *AtCTF7*-RNAi**. Columns indicate the mean of levels of expression, error bars indicate standard deviation.

**
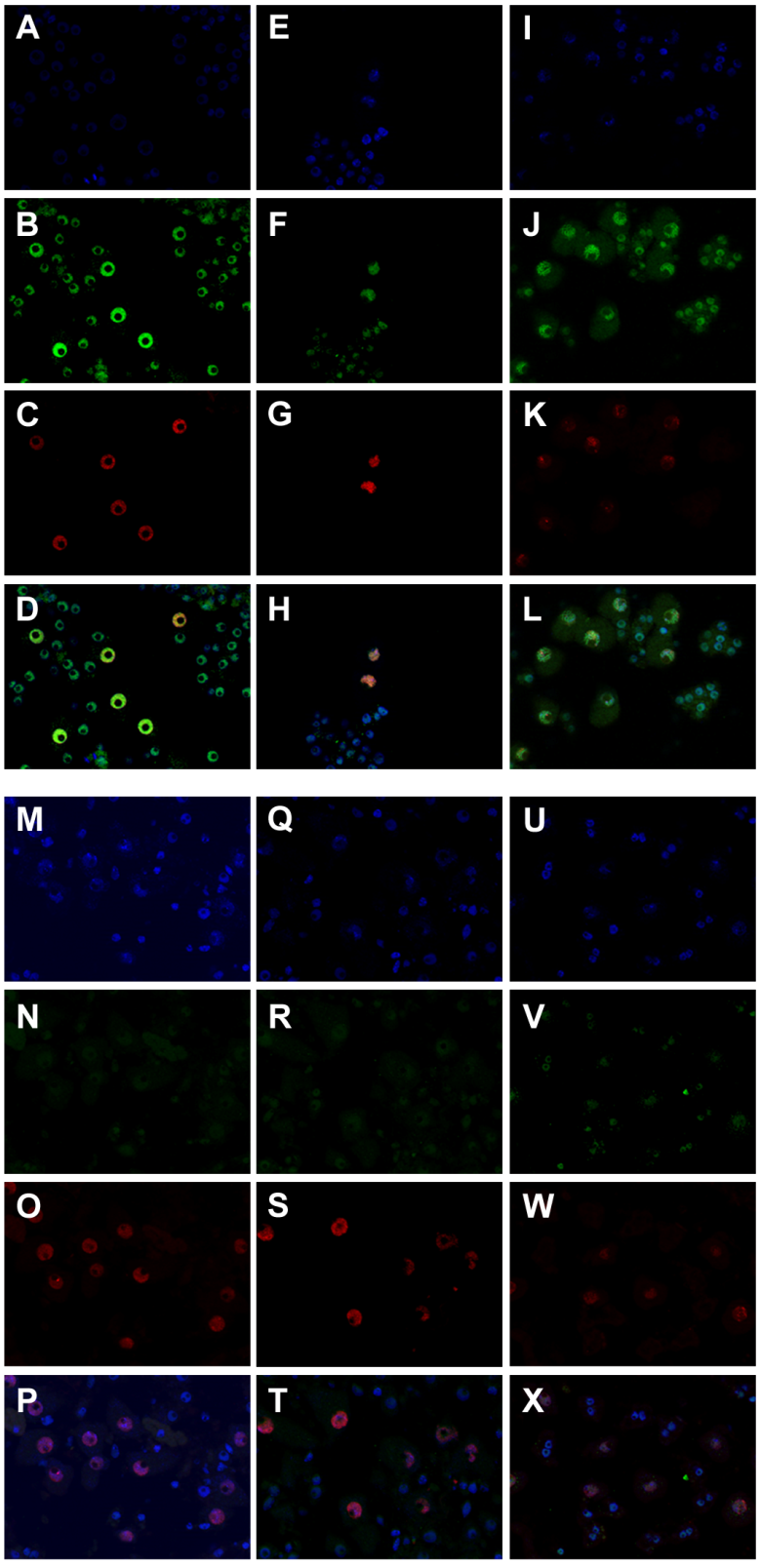
**

**Supplementary Figure 4: Raw pictures of AtSCC3 and ASY1 immunostaining on untreated and treated *AtCTF7*-RNAi line.** This figure shows raw microscopic images of whole fields from which single meiocytes are shown in figure 6. AtSCC3 (green) and ASY1 (red) immunostaining of untreated (**A-L**), and treated (**M-X**), *AtCTF7*-RNAi male meiocytes. Chromatin is stained with DAPI (blue). (**A-D**, **M-P**) Early leptotene. (**E-H**, **Q-T**) Leptotene. (**I-L**, **U-X**) Pachytene.

| Primer name | Sequence 5’-3’ |
| --- | --- |
| F1rnaiXba1 | cggtctagaggatgcatcgaaaagacgctgaagaaaggat |
| R1rnaiBamH1 | gtgggatcccccaaatcgctctaatcccacaaacagctgg |
| F1rnaiXho1 | cggctcgagggatgcatcgaaaagacgctgaagaaaggat |
| R1rnaiEcoR1 | gctgaattcccccaaatcgctctaatcccacaaacagctgg |
| Fctf7-gus_HindIII | gaaagctttgaatcagcttctcacttactttttttggctc |
| Rctf7gusnls.BamH1 | gaggatcccctacctttctcttcttcttgggagaagaagaggagggc |
| GAPRTR | cagtcttctgagtagcagtgattga |
| GAPRTF | agcacgaatacaagtccgacct |
| Ctf7qRTF | gcgcagaatgtggagctaa |
| Ctf7qRTR | taacgatgcggttcttgatg |
| Rpkanibal | cttcttcgtcttacacatcacttgtc |
| Fpkanibal | tgctaatataacaaagcgcaagatc |
| Rocspkanibal | taaggatctgagctacacatgctcag |
| R pal | AGTCTTTGGCTTTGTGTCTT |
| F pal | tggactttggctacaccatg |

**Supplementary Table 1: List of primers used.**
